# Zolpidem-Associated Dermatitis Artefacta—An Unusual Cause of Cicatricial Alopecia

**DOI:** 10.3390/diagnostics15233074

**Published:** 2025-12-03

**Authors:** Martyna Sławińska, Krzysztof Sadko, Beata Zagórska, Małgorzata Sokołowska-Wojdyło

**Affiliations:** 1 Department of Dermatology, Venereology and Allergology, Faculty of Medicine, Medical University of Gdańsk, 80-210 Gdańsk, Poland; beatazagorska@gumed.edu.pl (B.Z.); mwojd@gumed.edu.pl (M.S.-W.); 2 Department of Psychiatry, Faculty of Medicine, Medical University of Gdańsk, 80-210 Gdańsk, Poland; krzysztof.sadko@gumed.edu.pl

**Keywords:** zolpidem, complex sleep-related amnestic behavior, dermatitis artefacta

## Abstract

We present the case of a 95-year-old female patient who presented with erosions and scarring of the scalp, accompanied by long-term hair loss. Due to multiple comorbidities, she was treated with several medications, including zolpidem for insomnia. Clinical examination revealed scars and ulcers in the parietal region, along with diffuse age-related hair thinning. Histopathological analysis demonstrated scarring alopecia with features of local trauma. This case illustrates a rare instance of secondary cicatricial alopecia associated with zolpidem-induced complex sleep-related amnestic behavior. Detailed information on the patient’s medication history was crucial for establishing the correct diagnosis.

**Figure 1:**
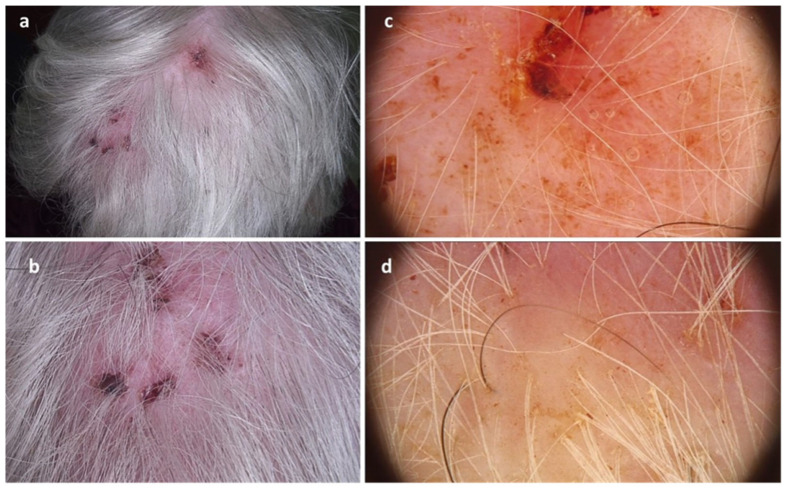
A 95-year-old woman presented with erosive and cicatricial lesions of the scalp with concomitant hair loss. The patient reports that the lesions have been present for a while, though the precise timeframe is unclear. In addition, she had a history of hypertension, diabetes type 2, hypercholesterolemia, hyperuricemia and varicose veins of the lower limbs. Due to the associated comorbidities, she was treated with amlodipine, perindopril, nebivolol, gliclazide, metformin, allopurinol, and rosuvastatin. Additionally, she reported long-term use of zolpidem, contrary to recommendations for short-term administration. The introduction of the drug overlapped with the onset of scalp problems. Clinical examination revealed the presence of cicatricial patches and ulcerations within the parietal region of the scalp (**a**,**b**), as well as diffuse age-related hair loss. Trichoscopy showed the presence of structureless red-yellowish areas corresponding to serohemorrhagic crust (**c**) surrounded with white structureless areas corresponding to fibrosis (**d**). Laboratory investigations did not reveal any abnormalities. During hospitalization, two punch biopsies were performed, followed by the wounds closure with sutures. Besides the patient’s good attitude to diagnostics procedures and excellent cooperation during and after the procedure, on the following night, she pulled the dressing and all the sutures out. In the morning, she had no recollection of her activities. Histopathological examination revealed the presence of scarring alopecia with the features of local trauma. Due to the suspicion of self-inflicted skin lesions, the patient was consulted with a psychiatrist, who diagnosed zolpidem-induced complex sleep-related amnestic behavior. Although the nocturnal behavior was directly observed only during hospitalization, it is plausible that similar episodes occurred previously but went unnoticed as the patient lived alone. Melatonin was introduced instead of zolpidem, which resulted in clinical resolution of the scalp lesions. Dermatitis artefacta (DA) or factitious dermatitis is a disease from the field of psychodermatology. It manifests with self-induced lesions which may concern skin, hair, nails or mucous membranes to satisfy an unconscious psychological or emotional need [1,2]. DA is more common in women, with an estimated mean age between 26 and 39 years [1]. Typical clinical presentation includes erosions covered with crusts, excoriations, ulcerations and erythema. In cases of chronic course, areas of hyperpigmentation and hypopigmentation, as well as scarring may be present, which within the scalp may lead to permanent hair loss (cicatricial alopecia) [2,3]. Sometimes, the patients report associated pain or itch. The most common location of skin lesions is the face, the limbs and the trunk, while the scalp is affected less frequently (4–13%) [2,4]. According to the literature, patients usually deny self-manipulations [1,3]. Zolpidem is a non-benzodiazepine agonist of the GABA-A receptor, mainly used in the treatment of short-term insomnia. In the spectrum of possible adverse effects of zolpidem, neuropsychiatric complications are mentioned, including complex sleep-related amnestic behaviors (e.g., making calls, writing e-mails, cooking or cleaning, or even car-driving) [4]. Similar complex sleep-related behaviors have also been documented with other pharmacological agents. These include antidepressants (amitriptyline, bupropion, paroxetine, mirtazapine), antipsychotics (quetiapine, olanzapine), and mood stabilizers (lithium). Nevertheless, most reported cases are associated with zolpidem and other agonists of GABA receptor. Women are more susceptible to this complication, due to slower drug metabolism [5]. Other recognized risk factors include endocrine disorders, concomitant use of sedatives or selective serotonin reuptake inhibitors, psychiatric comorbidities, and high or prolonged zolpidem doses, which could possibly contribute to this complication in the described patient. None of the other medications taken by the patient have been reported in the literature as potential causes of such complex sleep-related behaviors. Although no specific drug–drug interaction was identified as a direct cause, considering the patient’s polypharmacotherapy, age, and comorbidities, a cumulative effect cannot be excluded. To our knowledge, none of the previous reports described DA associated with zolpidem-induced complex sleep-related amnestic behaviors. The exact mechanism remains unclear, though a transient increase in frontal cerebral activity has been suggested [6]. Zolpidem’s high affinity for α1 GABA-A receptors and short half-life can impair immediate and delayed memory, potentially reducing cognitive control during peak drug levels and facilitating sleep-related behaviors [4]. To sum up, we report a rare case of secondary cicatricial alopecia associated with zolpidem-induced complex sleep-related amnestic behavior. Careful review of a patient’s medications may provide important diagnostic clues in similar cases. Zolpidem should be prescribed with caution, especially in geriatric patients with polipragmasia. The drug is indicated for short-time treatment (4 weeks) [7]. Clinicians should evaluate individual risk factors, comorbidities, and history of parasomnia. Close monitoring is essential, and the drug should be discontinued or switched if severe or unusual adverse effects occur.

## Data Availability

The original contributions presented in this study are included in the article. Further inquiries can be directed to the corresponding author.
